# Ribosome-Inactivating Proteins: From Plant Defense to Tumor Attack

**DOI:** 10.3390/toxins2112699

**Published:** 2010-11-10

**Authors:** Maddalena de Virgilio, Alessio Lombardi, Rocco Caliandro, Maria Serena Fabbrini

**Affiliations:** 1 Istituto di Genetica Vegetale, Consiglio Nazionale delle Ricerche, Bari, Italy; Email: maddalena.devirgilio@igv.cnr.it; 2 Istituto di Biologia e Biotecnologia Agraria, Consiglio Nazionale delle Ricerche, Milan, Italy; Email: a.lombardi@ibba.cnr.it; 3 Istituto di Cristallografia, Consiglio Nazionale delle Ricerche, Bari, Italy; Email: rocco.caliandro@ic.cnr.it

**Keywords:** ribosome-inactivating proteins, cancer immunotherapy, ITs, vascular leak syndrome, recombinant expression, fusion toxins, single chain antibody variable fragments, *Pichia pastoris*

## Abstract

Ribosome-inactivating proteins (RIPs) are EC3.2.32.22 N-glycosidases that recognize a universally conserved stem-loop structure in 23S/25S/28S rRNA, depurinating a single adenine (A4324 in rat) and irreversibly blocking protein translation, leading finally to cell death of intoxicated mammalian cells. Ricin, the plant RIP prototype that comprises a catalytic A subunit linked to a galactose-binding lectin B subunit to allow cell surface binding and toxin entry in most mammalian cells, shows a potency in the picomolar range. The most promising way to exploit plant RIPs as weapons against cancer cells is either by designing molecules in which the toxic domains are linked to selective tumor targeting domains or directly delivered as suicide genes for cancer gene therapy. Here, we will provide a comprehensive picture of plant RIPs and discuss successful designs and features of chimeric molecules having therapeutic potential.

## 1. Ribosome Inactivating Proteins and Plant Defense Mechanism(s)

Ribosome-inactivating proteins (RIPs) are toxins able to specifically and irreversibly inhibit protein translation. Most plants and bacterial RIPs, such as Shiga and Shiga-like toxins from the bacteria *Shigella dysenteriae* and the Shigatoxigenic group of *Escherichia coli* (which include other enterohemorrhagic *E. coli* strains), exert their toxic effects through binding to the large 60S ribosomal subunit on which they act as an N-glycosidase by specifically cleaving the adenine base A4324 in the 28S ribosomal rRNA subunit. This results in the inability of the ribosome to bind elongation factor 2, thus blocking protein translation [1,2]. RIPs are widely distributed in nature but are found predominantly in plants, bacteria and fungi. Besides their activity on rRNA, certain RIPs display a variety of antimicrobial activities *in vitro*, such as antifungal, antibacterial, and broad-spectrum anti-viral activities against both human and animal viruses, including the human immunodeficiency virus, HIV [3]. 

**Figure 1:**
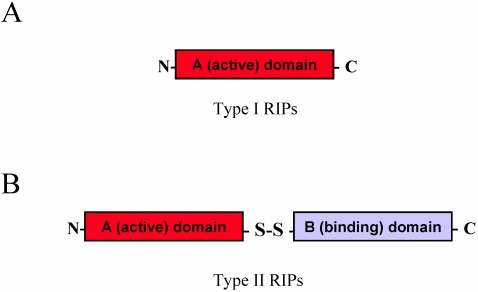
Schematic representation of the mature forms of Type I (**A**) and Type II (**B**) plant Ribosome-inactivating proteins (RIPs). Comparison between the mature forms of a Type I (RIP) (A), such as saporin, composed only of a catalytically active A domain, and that of a Type II RIP (B), such as ricin, in which the active domain is connected to a lectin binding B chain domain by a disulfide bond.

RIPs from plants have been classified into three main types: Type I is composed of a single polypeptide chain of approximately 30 KDa, Type II is a heterodimer consisting of an A chain, functionally equivalent to the Type I polypeptide [4], linked to a B subunit, endowed with lectin-binding properties [5] (Figure 1), while Type III are synthesized as inactive precursors (ProRIPs) that require proteolytic processing events to form an active RIP [6] and are not in use for therapeutic purposes. Besides their well characterized activity of depurinating ribosomes at the “sarcin/ricin loop” *in vitro* (see below), their physiological function(s) are not yet completely understood and the question as to why some plants should synthesize RIPs remains still open. Different RIPs have been reported from about 50 plant species covering 17 families. Some families include many RIP-producing species, particularly Cucurbitaceae, Euphorbiaceae, Poaceae, and families belonging to the superorder Caryophyllales [7,8].

Several biotechnological approaches have been applied to reveal a potentially important role of RIPs in plant defense ever since crude extracts of pokeweed leaves were first shown to have inhibitory activity against viral infections. To exploit antimicrobial activity, different RIPs, including pokeweed antiviral protein (PAP), trichosanthin (TC) from *Trichosanthes kirilowii* Maxim., and the antiviral protein from *Phytolacca* *insularis* Nakai, have been expressed in transgenic plants successfully, leading to resistance against various viral and/or fungal proteins [9,10,11,12]. 

Recently, two distinct saporin types from *Saponaria officinalis* L., saporin-L (leaf-like) and saporin-S (seed-like) isoforms were purified from the intra- and extracellular fractions of soapwort leaves. These isoforms differed in toxicity, molecular mass and amino acid composition. Differential expression of these saporin genes during leaf development and upon wounding and abscisic acid treatment has been described, indicating that different RIP isoforms may play diversified roles during plant stress responses [13]. The antiviral role of RIPs in plants is postulated on the basis of their enzymatic activity and selective compartimentalization [13,14,15,16]. RIPs may potentially inactivate ribosomes in the same cells in which they are synthesized and they are found sequestered into vacuoles, protein bodies, or cell walls [13,14]. It is suggested that when plants are wounded, for instance during viral infection, RIPs may be released from their intracellular compartments. This would prevent viral replication at an early stage by inactivating the cell protein synthesis machinery and leading to autonomous cell death [3,17]. However, the exact role of RIPs in planta still remains elusive, since also not all plant species express these toxins. In addition, most RIP-expressing plants present multigene families that seem to be under a clear selective pressure. A recent publication from the Craig Venter Institute revealed that whereas oil metabolism genes were found in single copy, the ricin gene family was even more extensive than previously thought, implying a strong selective pressure to maintain these ricin-like genes [18]. Among 25 geographically different castor bean plants, the presence of six ricin-like loci was confirmed, which shared 62.9–96.3% nucleotide identity with intact A-chains of the preproricin gene [19]. Replacement mutations preserved the 12 amino acids known to affect catalysis and electrostatic interactions of the native protein toxin, suggesting that functional divergence among alleles was only minimal. Nucleotide polymorphism was maintained but included an excess of rare silent mutations much greater than what would be predicted by a neutral equilibrium model [19]. 

Synthesizing an endoplasmic reticulum (ER)-targeted inactive precursor polypeptide is most likely the mechanism by which *Ricinus communis* L. cells can avoid intoxication by the endogenously synthesized toxin [20,21]. In addition, it has been shown that tobacco protoplasts can also safely synthesize the full length ricin precursor without any detrimental effect on host protein synthesis [22]. In contrast, the expression of an orphan secretory ricin toxic A chain (RTA) polypeptide results in the etro-translocation of RTA to the cytosol followed by inhibition of protein synthesis in the tobacco protoplasts, making RTA the most studied plant ER-associated degradation (ERAD) substrate, with the AAA-ATPase Cdc48/p97 being recently identified as an extraction motor for this toxin from the plant ER [22,23,24,25]. Little is known about the synthesis of Type-I precursor polypeptides in planta. Since several Type I RIPs are active towards “conspecific” ribosomes, and because of this observation, the inefficient targeting or translocation of Type I RIPs can potentially lead to self-intoxication, and therefore, mechanisms must be in place to prevent the unregulated accumulation of the active enzymes in the cytosolic compartment. In addition, this characteristic has led to the idea that these enzymes could play an important role in blocking the spread of certain pathogens by causing the death of infected cells [26,27]. Following the local suicide hypothesis [15,28], plant cells undergoing plasma membrane breaching by a virus would allow entry of apoplast-located toxins. This localized cell death would concomitantly block replication and the systemic spread of the virus load throughout the plant. In such a model, prior accumulation of the RIPs within the apoplast would be crucial. However, this mechanism was criticized because protein synthesis in damaged cells would be stopped during viral infection [6]. As an alternative, it has been suggested that specific mechanisms might regulate the access of a particular RIP to cytosolic ribosomes only when the plant cell becomes infected. The Iris RIPs, as an example, protect plants from local but not from systemic infections, indicating that their antiviral activity is effective only in the initially infected cells [29]. Specific signals may lead to a change in the subcellular localization of the toxin, or to the degradation of a putative RIP inhibitor [30]. One of these mechanisms might be embedded in the plant signal peptide, as we have recently demonstrated in the case of saporin [31] where cleavage of the signal peptide was found to represent an activation step in the saporin biosynthetic pathway. *In vivo*, mutations affecting signal peptide cleavage could clearly reduce (although not fully eliminate) host cell toxicity.

Stress-sensitive abortion of co-translational traslocation may provide a novel pre-emptive quality control pathway to regulate protein entry into the secretory compartment [32] and would lead in the case of saporin to activate the newly synthesized saporin precursor, allowing for its partial insertion into the ER to undergo signal peptide cleavage, and thus toxin activation.

## 2. Biochemical Characteristics

### 2.1. RIP Toxic Domains: Saporin Isoforms, RTA and 3-D Structures of Type I RIPs

**Figure 2:**
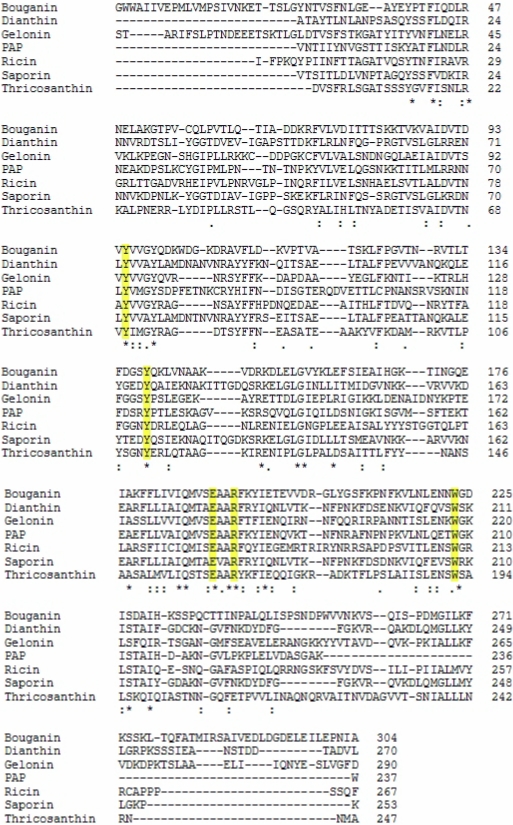
Amino acid sequence alignment of different type I RIPs compared to Ricin (A chain).

**Figure 3:**
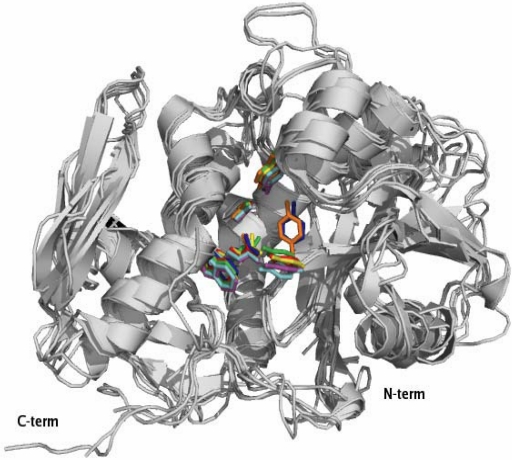
Three-dimensional structures of different Type I RIPs and ricin A chain. Superimposition of secondary structure elements of Ricin A chain (PDB code 1J1M), Thricosanthin (PDB code 1J4G), Saporin (PDB code 1QI7), Dianthin (PDB code 1RL0), PAP (PDB code 1PAF), Bouganin (PDB code 3CTK), and Gelonin (PDB code 3KTZ). Five conserved residues in the active site clefts corresponding to Saporin residues Tyr72, Tyr120, Glu176, Arg179, Trp208 are highlighted in magenta while the other colors refer to RIPs as following: Ricin in blue, Thricosanthin in red, PAP in green, Bouganin in yellow, Gelonin in orange. Crystal structures with high homology to the RIP sequences in Figure 2 have been superimposed by the SSM algorithm and this figure was produced by PyMOL.

Saporin collectively identifies a family of RIP isoforms that accumulate in different soapwort (*Saponaria officinalis* L.) tissues. Mixtures of closely related isoforms and several cDNA and genomic clones have been isolated. SO6 saporin or saporin-6 represents the major HPLC peak (peak 6) of purified seed protein and constitutes about 7% of the total proteins. For a comprehensive detailed description of the saporin isoforms investigated so far, please refer to [33]. Seed protein sequencing revealed heterogeneity at two positions, with either an aspartic or a glutamic acid in position 48, and either lysine or arginine present in position 91, indicating that the SO6 peak contains a set of closely related saporin isoforms. In fact, RP-HPLC analysis confirmed the presence of at least three different isoforms in SO6 preparations while recombinant expression of single seed-like isoforms demonstrated the same RIP activity, except for a leaf-derived isoform [34]. While some characteristics of the saporin proteins, such as key catalytic residues and overall three-dimensional fold, are shared with RTA and the other known crystalized RIPs, other biochemical features clearly differ among Type I plant RIPs and RTA. The sequence identity is low between RTA and Type I RIPs; RTA has only two lysine residues while lysine residues can account for up to 10% total amino acids in Type I RIPs. Indeed, amino acid sequence among type I RIPs and RTA may vary widely as can be seen by the alignment of some selected Type I RIPs in Figure 2, despite that all the crystallized RIPs have been shown to share a common three-dimensional fold, as can be estimated by the superimposition of the 3D structures of several Type I RIPs and RTA (Figure 3).

Only 22% of residues are conserved between RTA and saporin SO6, about 15% are shared between the latter and TC, while RTA and gelonin from *Gelonium multiflorum* A. Juss. share approximately 30% sequence identity [35]. On the contrary, a high degree of sequence identity (about 80%) is found between saporin SO6 and dianthin from *Dianthus caryophyllus* L., both of which are synthesized by plants belonging to the same subfamily of the Caryiophyllaceae family. The three-dimensional structures of RTA [36] and different Type I RIPs, including PAP [37], TC [38], gelonin [39], seed saporin SO6 [40] and, more recently, dianthin [41] have been determined, and demonstrate that RTA and Type I RIPs all share the common “RIP fold” characterized by the presence of two major domains: an N-terminal domain, which is mainly beta-stranded, and a C-terminal domain that is predominantly alpha–helical. Insertions and deletions, as compared to PAP, Momordin from *Momordica charantia* L. and RTA, lie mainly in random coil regions. Several residues are highly conserved among RIPs, including Tyr80, Tyr123, and the key active site residues Glu177, Arg180, and Trp211 of RTA (Figure 2 and Figure 3).

In Figure 2, residues highlighted in yellow correspond to conserved residues present in the active site (Tyr72, Tyr120, Glu176, Arg179, and Trp208 in Saporin). The sequence identities between Ricin A chain and the Type I RIPs analyzed are the following: Dianthin 19%, PAP 29%, Bouganin 29%, Thricosanthin 35%, Gelonin 30%. The sequence alignments have been performed with ClustalW. Asterisks indicate other conserved residues.

### 2.2. Interaction with Substrates and Catalytic Activity

The depurinating N-glycosidase mechanism of RTA is well understood. The target adenine in the substrate (28S rRNA) is inserted inside the catalytic cleft, with the aromatic ring becoming sandwiched between Tyr80 and Tyr123 with Arg180 partially or fully protonating N3 of the ribose ring, thus inducing a positive charge stabilization of the intermediate ribose by Glu177. Then, a water molecule is activated, probably by Glu177 [42], inducing nucleophilic attack to the N9-C1 glycosidic bond linking adenine to the ribose ring, then finally releasing free A4324. For most of the 3D structures, the Tyr80 aromatic ring is almost parallel to that of Tyr123/120, as required to form a stack with the adenine of the substrate, while for Ricin and gelonin Tyr80 is oriented in such a way that the hydroxyl group forms a hydrogen bond with the Gly121 carbonyl group (note the blue and orange residues, respectively, in Figure 3). For all the considered 3D structures, crystallographic analysis revealed a higher thermal parameter for the first catalytic residue with respect to the other conserved residues, indicating that it could work as a moving door for the adenine entering in the catalytic site [43]. 

Most of the secondary structural elements are comparable and superimposable between RIPs, including the catalytic cleft, as seen above, while the deviations are seen mainly in some surface-located loop regions. For instance, the loop connecting strands beta-7 and beta-8 located at the C-terminus is variable in length among RIPs, being very short in saporin SO6 [40] and in dianthin 30, but longer in PAP and RTA. This region contains several lysine residues, which seem to be involved in the molecular recognition of the ribosome. The reduced length of this loop could determine an increased accessibility to the substrate for both saporin and dianthin. The interaction between RIPs and the ribosomal proteins is essential to achieve optimal enzymatic activity; RTA can be cross-linked to mammalian ribosomal proteins L9 and L10e [43], while PAP recognizes L3 [44]. Chemical cross-linking studies suggest that at least one 30 kDa ribosomal protein from the 60S yeast ribosomal subunit comes into contact with saporin [45] by a region within the C-terminus which includes three lysine residues in positions 220, 226 and 234 [40]. Also in TC, three key basic residues (Lys173, Arg174 and Lys177), located at the C-terminal domain, are involved in binding to ribosomal proteins [46]. A recent study confirms that electrostatic interactions play a crucial role in facilitating several RIPs, endowed with a net surface positive charge, in finding their ribosomal target sites [47], since the latter exhibit a negative electrostatic potential, arising from both the negatively charged phosphodiester backbone and from conserved solvent-exposed acidic patches on the ribosomal proteins. In terms of their catalytic activity, RIPs were officially denominated “rRNA N-glycosidases” (EC 3.2.2.22) [48] as they removed the specific adenine residue (A4324 in rat liver rRNA) located in the universally conserved GAGA-tetraloop, also known as the alpha sarcin/ricin loop, present in 23/26/28S rRNA [49]. Some RIPs, however, have been reported to remove more than one adenine residue per ribosome [50], thus also possessing a polynucleotide: adenosine glycosidase (PNAG) activity [51] and, as better detailed below, they have been also reported to depurinate DNA [52] and other polynucleotides. The RIP active site includes a dozen of residues that are conserved among RIP family members, which include Tyr72, Tyr120, Glu176, Arg179, and Trp208 in saporin-6 (see Figure 2), and systematic studies were performed by mutating these residues, shedding light on their contribution to the catalytic activity of RTA [33] and Type I saporin [52]. Tyr72 mutation has a stronger impact on saporin-6 catalytic activity than Tyr120 mutation. Both Glu176 and Arg179 in saporin-6 (and Glu177 and Arg180 in RTA) are thought to be directly involved in catalysis. However, while the Glu176 mutant was 20-fold less active than wild-type in inhibiting translation in a reticulocyte lysate, the Arg179 mutant was 200-fold less active [52]. A complete loss of *in vitro* and *in vivo* saporin cytotoxicity can be achieved when Glu176 and Arg179 are mutated to lysine and glutamine residues, respectively. This double mutant (termed KQ) is, indeed, devoid of the detrimental effects associated with RIP expression in several hosts [31,53,54]. Interestingly, mutation of Trp208 in saporin did not impair its *in vitro* enzymatic activity and cytotoxicity [52] but this same residue was seen to be crucial for the structural integrity of PAP [55].

### 2.3. Extra Enzymatic Activities

More than 50 RIPs have been reported to depurinate other nucleic acids such as poly(A) RNA, herring sperm DNA, and capped RNAs and SO6 to depurinate a variety of polynucleotides [50,51,56,57,58]. However, the activity of seed-extracted SO6 was found to vary between different batches or experiments when poly(A) or viral RNAs were used as substrates [51], while recombinant single saporin isoforms [34] were instead found essentially inactive on poly(A) and viral RNAs, but active on rRNA at pH 4.0 [51]. In addition, it was also reported that some RIPs could hydrolize single-stranded and double-stranded DNA [52,55,57,58] in certain conditions. A comparison of the PNAG activity of bouganin, lychnin, dianthin 30, PAP-R, mormordin I, RTA and saporin-6 indicated that saporin-6 released the highest number of adenine molecules from rat ribosomes and poly(A), while its efficiency was similar to dianthin 30, bouganin and PAP-R on herring sperm DNA. At the same time, saporin-6 was found to be the most active toxin in inhibiting protein synthesis. A detailed structural analysis of the N- and C-terminal domains indicated that the efficiency of saporin-6 on various polynucleotides could be ascribed to a negative electrostatic surface potential at the active site and several exposed positively charged residues in the region around that site. These two conditions, which are not present at the same time in the other examined RIPs, would guarantee an efficient interaction with the substrate and efficient catalysis [59]. The debate is still open as to whether or not RIPs possess enzymatic activities other than N-glycosidase or PNAG, such as RNAase and/or DNAase activities [6]. Some RIPs have been even reported to have superoxidismutase (SOD) [60] and ricin A phospholipase [61] activity. Interestingly, although SOD activity reported for camphorin (from *C. camphora*) has not been independently confirmed, a 26-KDa RIP-like protein from *Nicotiana tabacum* has shown SOD activity in addition to an N-glycosidase activity [62]. In plants, both enzymatic activities may play a central role in defense response mechanisms and therefore it was tempting to speculate that the plant may differentially switch these activities, exerted by the same protein, upon specific environmental/biological requirements. The strong spatial similarities of type I RIPs, as shown in Figure 3, might suggest that different specificities/enzymatic activities may only reside in a limited number of polypeptide areas and a rigorous assessment of whether the latter may contribute to altered activity would require either site-directed mutagenesis or protein domain swapping experiments.

In conclusion, the biological significance of the activity against rRNA at sites different from the alpha-sarcin one and on substrates other than the ribosome (DNA, viral RNA, poly(A), RNA), remains to be firmly established. Like RTA [48,49], seed extracted saporin has been recently found to release a single adenine from 80S ribosomes [63]. In addition, when mamalian cells were exposed to seed-extracted saporin and rRNA was extracted from intoxicated cells, rRNA was found to be depurinated at a single site, most likely the one corresponding to the alpha sarcin-ricin target [64]. Removal of a second adenine residue or more adenine residues has also been reported for saporin-6 and saporin L1, respectively [59,63]. Analysis of the *in vivo* activity of other saporin isoforms (such as L1) [63] will be required to assess whether multiple depurination events play any major role in the mammalian cell’s intoxication process. In addition to the PNAG activities reported above, concerning the DNase-like activities [52,57] proposed for the type I RIPs, several pieces of evidence indicate that the DNase activity associated with ricin, saporin (and possibly with other type I RIPs) may be instead due to nuclease contamination [6,54,65,66].

## 3. RIP Intoxication Routes in Mammalian Cells

**Figure 4:**
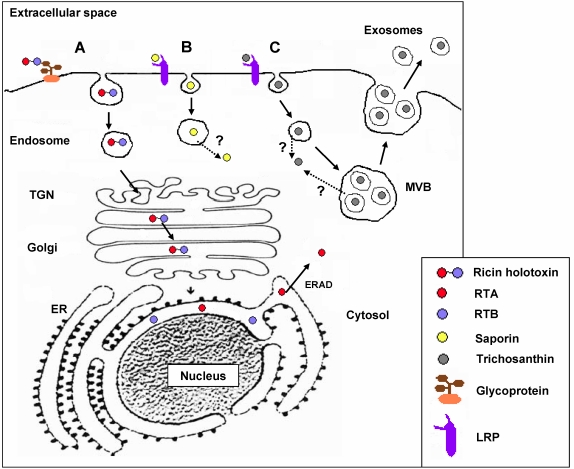
Schematic representation of the intoxication pathways followed by Ricin (**A**), saporin (**B**) and trichosanthin (**C**). (**A**) Ricin binds to cell surface glycoproteins and after endocytosis is transported through endosomes to lysosomes (not indicated) or following endosomal sorting steps to the *Trans* Golgi Network (TGN) [81] and then retrogradely transported from the Golgi complex (passing through sulfation compartments) to the ER [82], where the A chain retrotranslocates to the cytosol via ERAD pathways: RTA has been shown to interact with negatively charged lipid vesicles and with ER membranes, undergoing a conformational change making it a better substrate for the ERAD system [33,83]. The molecular details of cytosolic entry via an ERAD-specific membrane E3 ubiquitin ligase have also been revealed showing how RTA may avoid ubiquitylation and proteasome degradation [84]. Finally, after escaping degradation RTA may refold in the cytosolic compartment also thanks to the presence of host cell chaperones [85]. (**B**) Saporin binding to cell surface is at least in part mediated by low-density lipoprotein receptor-related protein (LRP) and after being endocytosed, the toxin reaches the endo-lysosomal compartment from where it is delivered to the cytosol following as yet unidentified pathway(s). (**C**) TC binding to cell membranes can also be mediated by LRP members and can be delivered to multivesicular body (MVB) and in part incorporated into the intraluminal vesicles of this organelle. Fusion of the MVB to the plasma membrane allows release of the intraluminal vesicles into the extracellular space where they diffuse and can target other syngeneic or allogeneic cells through unknown mechanism(s).

Ricin dimers efficiently enter target mammalian cells by receptor-mediated endocytosis, thanks to the presence of the lectin B domain that binds to exposed galactose residues, then the dimers undergo retrograde transport from the Golgi complex to the ER [21,67] where the catalytic A chain (RTA) is most likely reductively separated from the cell-binding B chain domain [68]. Free RTA then mimicks ER-associated degradation (ERAD) substrates to enter the cytosol [67] and may escape degradation also due to its low lysine content [69], thus inactivating cytosolic ribosomes and finally leading to apoptotic cell death (see Figure 4). Much less attention has been devoted to the study of the pathway(s) followed by saporin, and other Type I RIPs in general, during the mammalian cell intoxication process. For instance, saporin cytotoxicity varies in a wide range, with concentrations inhibiting protein synthesis by 50% (IC_50_) changing from nanomolar to micromolar, depending on the cell lines investigated [33]. The alpha2-macroglobulin receptor/low-density lipoprotein receptor-related protein (LRP1) binds saporin *in vitro* and mediates saporin internalization in human monocytes and in fibroblasts [33,34]. The LDL receptor family includes seven closely related family members: LDLR, the very-low-density lipoprotein (VLDL) receptor, apoE receptor 2, multiple epidermal growth factor-like domains 7 (MEGF7), glycoprotein 330 (gp330/megalin/LRP2), LRP1, and LRP1B that have been shown to be promiscuous in ligand binding [70]. LRP1-mediated saporin endocytosis in human promyelocytic U937 cells was also demonstrated by LRP downregulation, nicely paralleling resistance to saporin and to a urokinase–saporin conjugate [33], while displacement of iodinated LRP1-receptor associated protein (RAP) with saporin in these cells was also independently demonstrated [71]. Cytotoxicity of both saporin and an ATF-saporin chimeric fusion (see below) could be competed with large excess of antigen-purified LRP1 polyclonal antibodies in U937 cells [72]. In addition, LB6 fibroblasts transfected with the human receptor for urokinase were investigated to follow the internalization route of a model urokinase–saporin conjugate, demonstrating after cell surface binding to the urokinase receptors, again, a clear role for LRP1 in mediating the chimeric conjugate internalization [73]. However, when mouse embryonic fibroblasts (MEF-2) derived from LRP1 knock-out mice were exposed to saporin, they showed 10-fold less sensitivity to the toxin, as compared to MEF1 cells carrying both LRP1 and low-density lipoprotein receptor (LDLR) [64]; this possibly indicatES not only a role for LRP1 but most likely for LDLR, as well, in mediating saporin internalization in mouse fibroblasts. After being endocytosed saporin and RTA follow different intracellular routes: while RTA cytotoxicity can be blocked by the fungal inhibitor Brefeldin A (BFA) and can be enhanced by the addition of a C-terminus KDEL, ER-retrieval motif [33,74], neither the Golgi disrupting drug BFA nor addition of a KDEL sequence had any significant effect on saporin cytotoxicity [64]. To detect a functional role for ERAD in the intoxication path of exogenously applied saporin, toxin dose response curves were compared in wild-type and mutant CHO cell lines, exhibiting defects in the ERAD pathway affecting a number of ER-translocating toxins including ricin, but no differences could be detected, while evidence for escape from endosomal compartment(s) both for saporin and for a urokinase-saporin conjugate was provided [75]. The high cytotoxicity towards mammalian cells of chimeras containing saporin or other type I RIPs clearly indicates that these toxins have the ability to reach the cytosol after being internalized [76]. Thus, although different lines of evidence indicate that saporin and other type I RIPs can efficiently cross cellular membranes, the site(s) and mechanism of translocation might be quite different from those used by the catalytic subunits of type II RIPs. The intoxication pathway followed by TC (which behaves as an invasive toxin that targets syncythiotrophoblasts, macrophages, and T-cells) has been recently investigated [77]. TC also binds cell surface receptors belonging to the LDL-related receptor family, and its known *abortifaciens* and renotoxic actions are assumed to be caused by LRP1-mediated uptake in trophoblasts and by LRP2/megalin-mediated uptake in proximal tubule epithelial cells [78]. In agreement with the latter observations, Jurkatt-T cells (which do not express members of the LDL receptor family) are essentially resistant to free TC but turned sensitive to the TC-loaded vesicles found secreted by at least two target cell lines, JAR and K562 where part of the endocytosed TC was found incorporated into “pomegrenade” vesicles, deriving from multivesicular body (MVB) membranes, and was then secreted upon fusion of the MVB with the plasma membrane (Figure 4), targeting both syngeneic and allogeneic cells (Jurkatt-T cells). Whether other Type I RIPs are also able to exploit this exosome-mediated intercellular trafficking route remains to be clarified. Since some gelonin chimeric fusions are able to trigger mammalian cell death following an autophagy pathway [79,80], it would seem important to verify whether the latter toxin would also be able to hijack this novel exosome-mediated pathway. Finally, much work is required to elucidate the intracellular pathways followed by the different chimeric fusion toxins, in order to possibly potentiate their effects (see below). 

**Figure 5:**
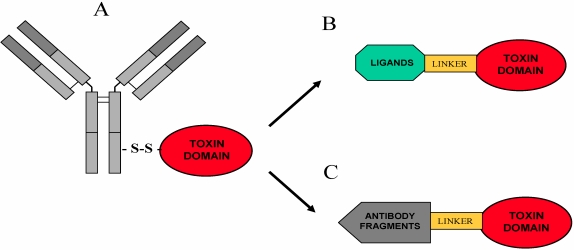
From chemically-conjugated immunotoxins to recombinant fusions. Schematic representation of a classical immunotoxin (IT) in which a monoclonal antibody is joined via a disulfide bond to a toxin domain (**A**) and recombinant fusions between sequences coding for (**B**) ligands (such as growth factor domains, hormones, autocrine factors) or (**C**) antibody fragments (single-chain antibodies, Fab fragments or disulfide-stabilized Fv) link to the toxic moieties via different Linker peptides.

The oxidizing potential of endosomes and lysosomes has been recently investigated in Prostate carcinoma cells (PC3) to check if the latter would limit the intracellular cleavage of disulfide-based antibody-drug conjugates, by a group in Genentech (Roche): Herceptin, approved by the Food and Drug Administration (FDA) in 1997 as a monoclonal anticancer therapeutic, was derivatized and conjugated via a disulfide sensitive SSP linker to a fluorescent rhodamine red dye. The latter has a quenched emission in a dimerized form and would emit only in a reduced monomeric form. Using a Green Fluorescent Protein (GFP) redox sensitive variant, roGFP1, which they appended as a tracer tag to various markers of organelles, such as Lamp2a for lysosomes or Calnexin for the ER or to the Transferrin receptor for labeling recycling endosomes, they evaluated that among internal membranes/organelles only the mitochondrial matrix showed a net reducing potential, questioning the use of reducible disulfides in the immunoconjugate design (see below, Figure 5) [86]. However, for certain ricin immunoconjugates it was suggested that not only the ricin holotoxin, but also the latter would be most likely reduced in the ER by Protein Disulfide Isomerase (PDI) coupled to a thioredoxin reductase activity [87].

## 4. Historical Uses of Plant RIPs

For many years, RIPs have found practical applications in different fields, such as in agriculture and in medicine. In agriculture, the effect of RIPs on plant viruses—the first to be known, led—as described above, to transformation of plants with RIP genes, successfully improving their resistance to viral infections [3], despite high-level expression of PAP being harmful to transgenic tobacco [9]. The antiviral activity against animal viruses has led to numerous studies on the effect of RIPs, especially concerning PAP [88] and TC [89], in HIV-infected cells, hoping for their possible use as an adjuvant in AIDS therapy. Although the replication of HIV could be inhibited by several RIPs, the few clinical trials gave disappointing results, revealing heavy side effects [90]. 

In old traditional Chinese medicine, extracts of the roots of the Cucurbitacea *Trichosanthes kirilowii* Maxim. have been extensively used to induced abortion, an effect due to the RIP TC [91], which is highly toxic to trophoblast cells [78,92].

Several type II RIPs are potential biohazards, and from time to time there have been reports of accidents due to ingestion of toxin-containing seeds or roots, causing severe intoxications which could not be effectively treated. Furthermore, ricin has long been considered as a possible weapon for warfare and terrorism attacks because of its high systemic cytotoxicity, ease of production and its prevalence: for these reasons ricin has been classified as a level B biothreat by the Centers for Disease Control [93]. The aim of several studies has been to detect and quantify ricin by highly sensitive methods, able to give rapid results if the diffusion of the toxin were suspected [94,95]. Moreover, to provide an immune protection against ricin, an intensive search for neutralizing antibodies has been performed [96]. Recently, a recombinant vaccine (RiVax) has been developed and found to be safe and highly immunogenic in mice, rabbits and humans [97,98].

Besides those reported above, we find that the most promising applications of RIPs in experimental medicine regard their use as targeted chimeric molecules chemically conjugated or especially as recombinantly expressed genetic fusions to different ligands, such as growth factors or antibody fragments, capable to deliver them to cells to be selectively eliminated (Figure 5). 

## 5. Immunotoxins and Targeted Chimeric Toxins

### 5.1. Overview

Despite progress in proteomics and bioinformatics approaches to characterize cell-surface antigens and receptors on tumor cells, it still remains difficult to identify novel tumor markers to generate cancer vaccines or monoclonal antibody therapeutics. A few targeted monoclonal antibodies have been approved by FDA to date for immunotherapy of malignant diseases with an average success rate of 30% [99]. To increase the toxin killing potential investigators made use of available target specific monoclonal antibodies which were derivatized to include a SS bond in order to allow chemical conjugation to the protein toxin of choice, leading to the construction of the chemically constructed immunoconjugates (Figure 5), also-called “Immunotoxins” (IT) (which will be the main subject of one of the next “Toxins” issues for readers interested in a deeper understanding of this vast and complex topic). For a more comprehensive view on ITs prepared using RIPs please refer to [100]. From these first chemically constructed ITs, research has naturally led from the pioneering work of Hudson and collaborators [101,102] to the most recent advances in design of recombinant single chain variable fragment (scFv) antibodies (which retain the immunoglobulin-derived domains that recognize antigenic epitopes) to scFv toxin fusions, the modern ITs [103] and to development of fully recombinant ligand toxins (Figure 5). Ontak or “Denileukin diftitox” is the first chimeric toxin which includes diphtheria toxin toxic domain and Interleukin-2, to be approved and commercially available for treating patients affected by recurrent T-cell lymphomas expressing CD25/IL-2 receptor [103,104,105]. 

Among critical factors that determine the successful delivery of an IT or cytotoxic chimera, in terms of specificity and cytotoxicity, is the antigen targeted on the tumoral cell surface. Ideally, a target should be almost exclusively found expressed on the tumor cells and should be capable of mediating internalization, after specific binding of the IT at the cell surface, in order to facilitate the access of the toxin moiety to the cytosol. However, genuine tumor-specific antigens are rare. Hence, an useful alternative is targeting antigens or receptors that are significantly over-expressed on tumor cells, so that peripheral toxicity is limited. 

The well documented immunogenicity of antibody-toxin constructs (see below) is due to the large size of the monoclonal IgG molecules, their mouse origin and the long circulation time, possibly enhancing antigenic properties of the toxin domains, as well. To circumvent these problems a number of molecular engineering approaches can be envisaged, in order to reshape the conventional antibody-toxin conjugate: antigenicity of the antibody moiety can be limited by humanizing the antibody and choosing smaller antibody formats containing only the variable domains, recognizing the antigenic epitopes of the heavy and light immunoglobulin VH and VL, linked together through a small peptide linker. Developing single chain antibodies adds flexibility in terms of the design of the engineered molecules (VH-VL or VL-VH orientation, type and length of the linker peptides, tandem sequence to allow for expression of dimeric (bivalent) *versus* monomeric molecules, bispecific constructs targeting more than one marker antigen, simultaneously. Improved tumor penetration is also expected due to the reduction in size. In fact, major concerns have been raised regarding plant toxins [105], particularly for their use in oncologic patients with solid tumor masses which may require prolonged treatment regimens [105]. In fact, it must be underscored that IT approaches would seem to be better suited for fighting hematologic malignancies, especially because of the different physiological/biologic barriers for local toxin concentration that solid tumors present against IT penetration and because patients with such malignancies are often immuno compromised [103,104,105]. Indeed, treatment of solid tumors may present potential bottlenecks due to the high interstitial fluid pressure gradient within the tumor generated by the hastily formed vascular architecture that limits the diffusion of large macromolecules, such as full-length antibodies, in the tumoral mass. This problem is also in part counteracted by using the smaller antibody formats, such as scFvs, for which several pharmacokinetics and biodistribution studies have been carried out [106,107,108]. Moreover, as stated above, single-chain antibodies may offer the possibility to reduce immunogenicity because of their smaller size, the absence of an Fc portion and, in particular, their more rapid clearance kinetics from the blood circulation.

### 5.2. RIP-Based Immunotoxins

Deglycosylated native ricin A was the first RIP to be conjugated to RFB4, an anti-CD22 monoclonal antibody, and examined in two phase I trials, one performed in 15 patients with refractory B-cell lymphoma where partial responses were achieved in 38% of evaluable patients, however, with three patients who made antibody against A chain, and a fourth who made antibody against both A chain and mouse immunoglobulin [109]. Vascular leak syndrome (VLS, see below) was dose-limiting for this RFB4 IT when administered to 26 patients whose B-cell lymphoma had relapsed after conventional therapies [110]. As compared to ricin-based ITs, saporin has been exploited relatively little as the toxin of choice for clinical uses, so far [100,109,111]. A small clinical study with an anti-CD30 monoclonal conjugated to the seed extracted saporin (BERH2-SAP) and administered to patients with advanced Hodgkin’s lymphoma, proved very encouraging [111]. Out of four patients treated with escalating doses of BERH2-SAP, one had a complete response and two had partial responses with a 75% to 25% reduction in the tumor mass. Notably, no serious drug-related toxicities were reported in this study; however, antibodies against both domains were raised in the treated patients. ITs based on saporin have been used by Flavell and coworkers for their clinical trials in adult and pediatric patients with hematological malignancies, their pre-clinical studies showed that an anti-CD19 IT BU12-saporin and an anti-CD38 IT OKT10-saporin [109] displayed selective antitumor activity both *in vitro* and *in vivo* against malignant target hematological cells. In addition, by using combinations of ITs targeting more than one cell surface marker antigen simultaneously, a significant synergistic therapeutic performance could be obtained in Severe Combined Immunodeficiency (SCID) mice model of leukemia and lymphoma [112]. An anti-CD19 saporin IT has previously also been evaluated in two separate Phase I studies in adults with B-cell follicular lymphoma and in children with relapsed and refractory B-lineage acute lymphoblastic leukemia (ALL). No major toxicity was encountered and none of the patients developed human anti-mouse antibody responses [109]. However, the intrinsic heterogeneity of all such chemical conjugates would have precluded their successful development into potential therapeutic molecules [104,109]. Recombinant approaches are now pursued also by Leukemiabuster charity, as well, to open the way to a new class of saporin-based therapeutic molecules against hematologic malignancies [54].

Some ITs have been produced but only using RIP domains expressed in bacterial hosts or, *vice versa*, by conjugating recombinantly expressed targeting domains to the native protein toxins. Among them, a conjugate of recombinant RTA with a monoclonal against the Transferrin (Tn) receptor [113], directed towards brain neoplasia and administered intraventricularly to patients, and a conjugate of anti-CD33 humanized monoclonal antibody with recombinant gelonin, endowed with antileukemic activity [114]. Recently, an antileukemia IT has been obtained with a single chain antibody expressed in the yeast *Pichia pastoris* derived from an anti-CD7 monoclonal antibody which was, nevertheless, chemically conjugated to RTA [115]. In addition, some other recombinant-based chimeras have been tested such as one between the N-terminus of the viral glycoprotein of vesicular stomatitis virus fused to RTA that was produced and then, nevertheless, chemically conjugated to human Tn and that showed a 15-fold increase in cytotoxic activity as compared to the parental IT [116]. A promising approach *in vivo* for therapy of transplanted organ rejection and graft-versus-host disease (GVDH) is to efficiently kill alloreactive cells by selective depletion of T cells, targeting Cytotoxic T-lymphocyte Antigen-4 (CTLA4/CD152) [117]. CTL4 is highly expressed on several hematologic malignancies and on activated T cells, responsible for transplant rejection and autoimmune diseases. Two phage display selected single-chain human antibodies, scFv83 and scFv40, recognizing two different epitopes on CD152 dimers, were chemically conjugated via a SS bond to seed extracted saporin and their efficacy *in vivo* in an acute mouse rejection model investigated. The inhibitory *in vitro* activities measured in reticulocyte translation after IT reduction were found to be similar for both IT, only about 3–5 fold less potent than saporin alone. CTLA4 surface expression is restricted to T cells and upon T cell activation dimers become rapidly internalized: cell killing activities were assayed against activated T cells and B lymphoblastoid cells, as allogeneic targets. The 83-saporin concentration causing 50% apoptotic death (AC50) of stimulated T lymphocytes was in the picomolar range. Since 83-saporin showed a stronger reactivity, the latter IT was chosen for *in vivo* administration to a mouse model carrying an implanted allogeneic endothelioma displaying T lymphoid cell inflammatory infiltrates. 4 µg 83-saporin IT was injected in a group of four mice at days 0, 1 and 2: while a marked infiltration was detected in tumors of control PBS-injected mice, scarce to moderate infiltration was observed in treated mice, with—most importantly—no signs of acute organ toxicity post-mortem [117]. Recently, an internalizing human anti ALCAM-scFv was also selected by phage display on whole tumor cells and then conjugated to seed saporin in a similar way, showing specific killing activity against target tumor breast cells [118]. These data demonstrated that the phage display techniques can be used to select scFv variants with an improved efficacy when fused to seed saporin, reducing the toxic side effects normally associated with this approach.

### 5.3. RIPs and Recombinant Fusions Expressed in Bacteria: Targets and Molecular Design

The use of plant-extracted RIPs as such or incorporated into immunoconjugates could be limited during preclinical and clinical testing by the presence of heterogeneous preparations with several immunologically distinct toxin isoforms which may also vary significantly in their catalytic activities [33], thus recombinant expression of single isoforms is advisable. The *Escherichia coli* bacterial system was the first one to be explored. The recombinant protein may be recovered as soluble material, or may accumulate inside inclusion bodies [33,109]. In this latter case, inclusion bodies must be isolated, purified and solubilized. The proteins are then extracted by denaturation and refolded in the presence of an appropriate redox buffer, while endotoxin contaminations may be a common problem of bacterial expression systems [119].

While RTA was successfully produced in bacterial cells [120] since it is not toxic towards prokaryotic ribosomes, in constrast, dianthin 32, which is highly similar to seed saporin, was found to be active on *E. coli* ribosomes and 500-times more active on yeast ribosomes, specifically depurinating 23S rRNA at a site which is equivalent to A4324 in rat 28S rRNA [121]. Therefore, saporin and other type I RIPs, such as PAP [122] and *Mirabilis* antiviral protein [123] and more recently luffin [124], being active also on *E. coli* ribosomes [121], required a tight regulated host/vector inducible system, such as the one used for the expression of dianthin and mature saporin isoforms [33,34]. Plasmid pET vectors used for these expressions contain the T7 promoter, and are used to transform *E. coli* strains (*i.e.*, BL21(λDE3)) containing the T7 RNA polymerase gene in the bacterial chromosome and placed under the control of lacUV5 promoter. Addition of isopropyl-D-thiogalactopyranoside (IPTG) induces expression of T7 RNA polymerase and hence of the recombinant toxin. A tighter control of T7 RNA polymerase expression is obtained using plysS-containing *E. coli* strains (*i.e.* BL21(λDE3)plysS, AD494(λDE3)plysS), which constitutively produce low amounts of T7 lysozime—a natural inhibitor of T7 RNA polymerase [125]—thus, avoiding any leaky accumulation of active RNA polymerase and, consequently, of the toxin. Indeed, by using this host, no saporin polypeptides were detected in the absence of the inducer, but a few milligrams of soluble protein per liter could be purified from bacterial lysates, part of the protein being accumulated as inclusion bodies [34]. Recently, a recombinant saporin bearing a C-terminal VSAV tetrapeptide (SapVSAV), recognized by a specific PDZ domain, has been expressed in the BL21(DE3) host. The yield of saporin in the soluble fraction was greatly enhanced when the corresponding PDZ domain was co-expressed. Surprisingly, the increase in saporin production was not due to protection from bacterial auto-intoxication but most likely from a stabilization effect of the PDZ domain on the toxin during biosynthesis. Interestingly, this recombinant saporin was not toxic to free ribosomes although fully active to human cancer cells [126]. Concerning Type I plant RIPs, only a few recombinant fusions have been produced until now.

An intriguing example concerning RTA fusions is a hybrid toxin expressed in *E. coli*, PE-RTA, in which the ADP-ribosylation domain of Pseudomonas Endotoxin A was replaced by the *N-*glycosidase domain of RTA. The latter chimera has been used essentially to investigate the intracellular pathway(s) of these toxins, demonstrating that cytotoxicity of PE-RTA was approximately two orders of magnitude less than either native PE or the ricin holotoxin, but that the addition of the KDEL tetrapeptide to its *C*-terminus (producing PE-RTA-Lys-Asp-Glu-Leu) increased cytotoxicity to the same level of the native toxins [127]. 

PAP has gained considerable interest for its use as either a ribosome inhibitory anticancer agent, or as a broad-spectrum antiviral agent (see above) and some efforts to obtain active PAP-based fusion proteins have been made. A recombinant chimera between PAP and Interleukin IL-2 was expressed in *E. coli* and the inclusion body fraction, mainly containing the recombinant protein, was isolated and a denaturation/renaturation process established to prepare the soluble fusion protein [128]. The fusion protein was as active as free PAP in inhibiting reticulocyte cell-free translation system while only the chimeric protein inhibited translation of the CTLL-2 target cell line which expresses IL-2 receptors [129]. On the contrary, in a more recent work, either mature PAP or the full-length precursor sequence containing both the signal peptide and the C-terminal propeptide were fused to the gonadotropin-releasing hormone (GnRH) to specifically target cells bearing the GnRH receptor, including several cancer cell types, and expressed in *E. coli* [129]. Binding and cytotoxicity of these two hormonotoxins were compared to a chemically-conjugated GnRH-PAP: surprisingly, although the conjugate bound specifically to and caused cell death via apoptosis in cells exposing the target receptors, no binding nor cytotoxicity was observed when the same cells were treated with the latter two recombinant fusions, possibly because both ends of the GnRH moiety are required for receptor binding, while only the N-terminus was left free in the recombinant chimeras [129]. 

Basic fibroblast growth factor (bFGF)-saporin was the first recombinant fusion chimera based on saporin expressed in *E. coli* shown to be highly selective and cytotoxic towards FGF2 receptor-expressing cells [130]. Moreover, recombinant basic FGF-saporin fusion also showed significant anti-proliferation activity when tested in animal models of human ovarian teratocarcinoma or melanomas in a combination therapy [131,132]. Another example of a fully recombinant saporin fusion is represented by an EGF receptor-targeted fusion between a heparin-binding epidermal growth factor (HEGF) and saporin, showing selective cytotoxic activity towards human breast carcinoma cells [133]. 

One of the fusions targeting the receptor of urokinase (uPAR) is ATF-SAP, which consists of the N-terminal portion (ATF) of the receptor binding domain of human urokinase (huPA) fused to a seed saporin isoform which has shown the potential to specifically kill tumor cells over-expressing huPA receptors [72]. uPAR focuses pro-uPA at the cell surface, increasing its rate of conversion into active uPA, a serine protease which converts plasminogen into active plasmin, leading to cell matrix degradation [134,135]. Human uPAR is over-expressed at the leading edge in several metastatic tumor cells, mediating tumor cell dissemination [136,137,138,139,140], and this is the reason why this receptor represents an important drug target [134,135,136,137,138,139,140]. An intriguing feature was that, in contrast with uPA, ATF-SAP did not require plasminogen activator inhibitors (PAI) to be internalized, indicating that saporin has the ability to trigger internalization of uPAR-bound ligands through LRP endocytotic receptors [70,71]. However, ATF-SAP expression into the bacterial host was problematic due to misfolding problems [72], thus hampering further pre-clinical and clinical investigations. Pseudomonas exotoxin A and diphtheria toxin are the most commonly used bacterial toxins in IT constructs [141] because they also inactivate protein synthesis by ADP ribosylation of elongation factor 2, thereby leading to cell death. DTAT is a diphtheria toxin fusion similar to ATF-saporin, produced by fusing a toxic fragment of the diphtheria toxin (DT) to ATF [142,143,144]. A bispecific IT, DTAT13, was then further engineered by adding the mature peptide of human IL-13 in between the ATF portion and a diphtheria toxin toxic moiety [145,146]. DTAT and DTAT13 could effectively kill tumoral cells in glioblastoma multiforme and acute myelogenous leukemia [146]. Efficacy of DTAT13 was significantly better in inhibiting a range of xenograft tumors and showed that DTAT13 was 160- and 8-fold less toxic compared to the parental fusion ITs, DTAT and DTIL13 [146]. Intracranial biodistribution studies revealed that DTAT13 was able to cross to the contralateral hemisphere, unlike DTIL13 but similar to DTAT. These studies showed that DTAT13 has properties encompassing those of both DTIL13 and DTAT and would warrant further consideration for clinical development [146]. However, in spite of the great promises shown by bacterial toxin-based chimeric proteins, their use also posed several obstacles that have limited their wider clinical application, since the toxin part of these fusion proteins may elicit a high degree of humoral response in humans [147]. Another bispecific DT recombinant IT targeting both CD19 B cell marker of leukemia/lymphoma and CD22 marker on B cell leukemias (which had a much better antitumor activity than a mixture of the two monospecific ITs), recently in Phase I clinical trials, encountered the same immunogenicity-related problems encountered with Ontak/denileukin [80,94]. Besides, in developed countries where people are immunized against diphtheria, serum will have circulating antibodies against diphtheria toxin [148]. 

The development of recombinant ITs has markedly affected the fields of targeted therapeutics, allowing different molecular design approaches, in order to engineer molecules with improved *in vitro* and *in vivo* stability and better functionality [149,150]. 

The Type I RIP gelonin is one of the most promising plant toxins used to construct several recombinant ITs expressed in bacteria. One anti melanoma IT was obtained by fusing the N-terminus of gelonin to a single-chain antibody directed against gp240, the high molecular weight glycoprotein which is specifically over-expressed in melanoma cells [79]. This IT was characterized by having a VL domain connected to the VH domain via an 18-amino acid flexible linker (218 linker: GSTSGSGKPGSGEGSTKG), enhancing proteolytic stability and causing reduced aggregation of scFvs when expressed in bacterial systems [151]. A G_4_S peptide linker [79,80] was used to connect the single chain antibody to the toxin domain, carrying a hexahistidine-tag for nickel affinity purification. IT was expressed in the thioredoxin-reductase minus *E. coli* AD494(DE3) pLysS strain, with yields around 0.7 mg per liter down to 0.2 mg of purified material, possibly the purification was too inefficient purification due to hindering of the hexahistidine-tag. The protein synthesis inhibitory activity of the fused gelonin was virtually identical to the non-fused toxin (around 100 pM), suggesting no significant steric hindrance of the gelonin active-site cleft is given by the single chain antibody domain. This would seem to contrast with studies using ricin A-chain-based IT, which required release from the protein partner to recover full biological activity [120,152]. The IT showed an IC_50_ value of 8 nM (*versus* 2000 nM of free gelonin in an *in vitro* cytotoxic assay on antigen-positive A-375 human melanoma cells), identical to the parental gelonin chemical immunoconjugate. Surprisingly, cytotoxicity was not mediated by apoptotic mechanisms. In addition, studies conducted on tumor-bearing nude mice showed that the tissue: blood ratio was highest for tumors with respect to other tissues at 72 h after administration of the IT, due to its relative rapid clearance from the circulation [77]. A more recent study provides a comprehensive examination of the effects of IT design on the ability of different constructs to target cells expressing the *Her2/neu* oncogene product, a 185 kDa transmembrane glycoprotein kinase, which is highly expressed in breast and ovary cancers [153]. By constructing ITs containing either a human single-chain antibody (scFv) C6.5 or the murine scFv e23 fused to recombinant gelonin (rGel) molecule, using the flexible G_4_S linker (L), the fusion construct C6.5-L-rGel was directly compared to e23-L-rGel and specific cytotoxic effects evaluated against Her2/neu-positive and Her2/neu-negative tumor cells. Both constructs retained the specificity of the parental scFv antibody, as well as the biological activity of rGel toxin and displayed similar cytotoxicities against different carcinoma cells. In addition, two different cleavable linkers Fpe linker: TRHRQPRGWEQL (a sequence deriving from Pseudomonas exotoxin A) and Ftd linker: AGNRVRRSVG (a sequence deriving from diphtheria toxin) containing sites proteolytically cleaved by furin, a protease found in recycling endosomes to Golgi, were also examined to check for a more efficient intracellular release of gelonin. The introduction of the cleavable linkers into the molecules resulted in a dissimilar sensitivity to protease cleavage, as compared to the first two constructs containing the L linker alone, although they all exhibited a very similar intracellular rGel release, cytotoxic kinetics, and induction of autophagic cell death. However, constructs carrying the L linker showed higher stability *in vitro* as well as in xenograft mouse studies where they were more efficient in tumor inhibition than the constructs containing furin sensitive linkers, thanks also to their higher stability *in vivo* [153]. A further gelonin-based chimera was generated by fusing the toxin to a B lymphocyte stimulator (BLyS), a member of the tumor necrosis factor superfamily of cytokines that selectively binds B cells and is known to promote B cell proliferation, activation and differentiation. BlyS receptors are over-expressed in a broad range of tumors, including multiple myelomas and several lymphomas, such as large B cell lymphoma, non-Hodgkin’s lymphoma and Burkitt’s lymphoma. The fusion toxin rGel/BLyS containing gelonin at the N-terminus, followed by the G_4_S linker peptide tethered to BLyS, was expressed in the bacterial system and was found to be highly cytotoxic against lymphoma cell lines, being rapidly internalized into target cells [154]. Differently from the gelonin-based chimeras described above, further studies provided strong evidence that its *in vitro* and *in vivo* cytotoxic effects could be mediated by canonical apoptotic mechanisms [155,156,157].

Unlike antiangiogenic drugs that inhibit formation of new blood vessels, vascular targeting agents (VTA) may occlude pre-existing blood vessels of tumors to cause tumor cell death through ischemia and extensive hemorrhagic necrosis. Among ligand-based VTAs, we may find vascular endothelial growth factor fusions (VEGF) to the plant toxin gelonin, ITs (monoclonal antibodies to endoglin conjugated to ricin A), antibodies linked to cytokines, liposomally encapsulated drugs and gene therapy approaches [158]. (VEGF)-gelonin is a highly selective carrier, targeting tumor endothelial cells, which showed promising applications. (VEGF)-gelonin fusions are able to inhibit tumor growth and metastasis dissemination, and recently, by a fed-batch cultivation method the investigators could achieve high cell densities, comparable to those obtained with the methylotrophic yeast strains (of about 40 OD/mL) with an overall production of a biologically active histidine-tagged VEGF-gelonin fusion in AD494 pLysS of 42.5 mg/L (compared to 1.6 mg/L produced using conventional flask cultivation methods) [159]. An Abrin-VEGF121 fusion protein was also recently obtained in *E. coli* in inclusion bodies, yielding both monomeric and dimeric forms [160]. Basic FGF, VEGF and EGF are relatively short protein domains which may not require special folding assistance and which do not need N-or O-glycosylation for being biologically active, and thereby, good yields may be obtained when expressed as fusion toxins in bacterial hosts. However, some fusions were quite difficult to produce in this system, such as the one between ATF and saporin due to misfolding and degradation of the human targeting domain [72].

## 6. Yeast/Eukaryotic Expression Systems

Differently from bacterial toxins-based fusion proteins which can be expressed at high yields in the bacterial system, only few recombinant fusions containing plant RIPs have been successfully produced using the bacterial hosts. Surprisingly, despite RTA could be produced in bacteria at high levels, recombinantly expressed fusions to RTA did not seem to be very popular [109]. Thus, development of eukaryotic production platforms for safe expression of RIP fusion proteins would be highly desirable, since low protein yields, endotoxin contaminations and inefficient folding of eukaryotic secretory domains used for targeting, as in the case of the ATF domain [72], represented potential bottlenecks in using prokaryotic expression systems. 

Human interleukin-2 was fused to RTA and constructs containing N-terminal IL2 and C-terminal RTA, or N-terminal RTA and C-terminal IL2, could be expressed in *Xenopus laevis* oocytes. The IL2 and RTA domains were joined by a peptide in which two different proteolytically-sensitive peptide sequences were utilized: one that forms the trypsin-sensitive disulfide-bonded loop in DT or a synthetic peptide containing factor Xa recognition site. Chimeras not pre-digested with factor Xa (but which contained the factor Xa target sequence) were not cytotoxic to CTLL-2 cells. Rather, they showed a stimulatory effect due to the IL2 moiety. In contrast, recombinant chimeric toxins containing the DT loop sequence were found cytotoxic to CTLL-2 cells [161]. Taken together, these data suggested that RTA-containing chimeras required intracellular proteolytic cleavage to allow release of the toxin moiety and would suggest that a non toxic precursor was expressed and secreted in the oocyte system. Secretory ATF-saporin could be expressed in micromolar amounts using the Xenopus oocyte expression system but only if the oocytes were co-injected with neutralizing anti-saporin antibodies, indicating that saporin was able to reach the cytosolic compartment, in this case [74].

In the last years, the methylotrophic yeast *Pichia pastoris* has been shown to be a suitable host for high-level expression of various heterologous proteins, especially endowed with clinical and potential high therapeutic value, expressed either intracellularly or in secreted forms [162]. Indeed, *Pichia pastoris* is a microbial host that recapitulates most of the co- and post-translational modifications during protein biogenesis and possesses an ER quality control system allowing only secretion competent polypeptide to reach the extracellular medium. This eukaryotic system was exploited to successfully express mature PAP, which was expressed as a precursor carrying the preproalpha mating factor leader sequence and secreted by the protease-deficient strain SMD1168H with average yields of 12 milligrams recombinant PAP (rPAP) per liter of culture supernatant. The secreted rPAP showed the same *in vitro* enzymatic activity and cellular anti-HIV activity of native PAP purified from Pokeweed, *Phytolacca americana* L. [163]. Interestingly, it was found that rPA expression was not toxic to the yeast cells, although this RIP should be able to inactivate both prokaryotic and eukaryotic ribosomes [9,12,109,121], thus suggesting that a rapid and/or very efficient secretion into the culture medium likely prevented rPAP from interacting with the yeast host ribosomes [163]. Neville's group pioneered the expression of recombinant IT fusions to DT in *Pichia pastoris* and found GS115 strain particularly tolerant to this bacterial toxin [54,164]. More recently, *Pichia pastoris* was also explored for secretion of saporin and saporin fusion chimeras [54], however, in contrast to what was found during PAP expression [163], SMD1168 strain was not able to induce this plant RIP. Moreover, independently from the strains used and the construct design, some host toxicity was always observed when inducing saporin expression, which was due to RIP activity, since the catalytically-inactive KQ saporin mutant (see above) showed no such toxicity when expressed under the same conditions. It is worth noting that saporin gene-codon optimization was essential to obtain clones expressing high-levels of active saporin in GS115 strain (up to 30 mg/L), presumably due to lower detrimental effects on the cell host protein synthesis machinery [54]. Although saporin was efficiently secreted, secretion efficiencies were lower when some fusion chimeras were expressed, a fraction of the latter being retained in the cell and degraded in the yeast vacuole [54]. Notably, secretion efficiency was found to strictly depend on the nature of the targeting domain fused to the toxin: while saporin recombinant ITs with different murine or human single-chain antibodies were mostly retained and degraded, the secretion efficiency of ATF-saporin chimera was comparable to that of saporin, indicating that a good secretory domain may be important to avoid misfolding and/or targeting to the vacuolar compartment [54]. Among the saporin recombinant ITs investigated, different construct designs were tested and the expression levels of the resulting saporin chimeras have been compared. Our results indicate that gene-codon optimization of the targeting domain seems also essential to obtain the latter in high yields [165],). In addition, we also used the 218 linker [151] between the variable VH and VL domains in the scFv design, finding it was much better than the canonical (G_4_S)_3_ linker [101,102] also during expression of saporin fusions to scFv in *Pichia pastoris*. However, when we tested the reverse configuration: VL-218L-VH, widely used in many toxin-based ITs [79,80], no fusion polypeptide could be induced. The highest secretion yields were obtained when the saporin toxin domain was instead placed at the N-terminus of the scFv but in this case no cytotoxicity data are yet available, thus, we can not exclude that this particular fusion might be non toxic [165].

## 7. Immunogenicity and Non Specific Toxicity Issues

A major drawback when using murine targeting domains, as well as plant or bacterial toxins as therapeutic agents, is represented by their potential immunogenicity after repeated administrations. The development of ITs containing humanized antibodies or antibody fragment lacking Fc portions may in part resolve these problems by avoiding the induction of human anti-mouse antibodies (HAMA) directed to the Fc murine fragments in treated patients [166,167,168]. Moreover, the presence of a bacterial or plant toxin can trigger the formation of neutralizing antibodies, hindering their efficacy. In patients with B- or T-cell malignancies, the formation of neutralizing antibodies is less frequent because of their immuno-suppressed state; in contrast, in patients with solid tumors, antibody responses are frequently detected as early as a few days after the first treatment regimen, preventing re-administration of the ITs [169,170]. Many efforts are being made to decrease immunogenicity of the toxin moiety; one possibility explored is masking of the therapeutic molecules by use of polyethylenglycol (PEG) derivatization techniques [171]. An impressive result was obtained recently by Onda and coworkers, who identified the seven major immunogenic B-cell epitopes in a truncated form of *Pseudomonas aeruginosa* exotoxin A (PE38) [172]. A total of eight amino acids containing large bulky hydrophilic side chains have been replaced with smaller polar residues within these epitopes, resulting in a new toxin endowed with much less immunogenicity than the parental one, without any loss of cytotoxic activity also when recombinantly fused to either an anti CD22 variable fragment (which was tested both *in vitro* and in mice model of lymphomas [173]) or used in a bispecific anti CD22 and anti CD19 IT construct carrying an ER-retrieval motif (2219KDEL7mut) [80]. In the latter case, serum from mice immunized with multiple injections of 2219KDEL7mut had 80% less antitoxin antibodies than mice immunized with the non mutated IT [80]. Interestingly, human IT treatment induces human antibodies against the same seven immunogenic epitopes identified in the mouse sera [172,173], thus confirming that the murine model is an acceptable immunogenicity model also for humans.

Concerning the plant toxins, the C-terminal domain (residues 203–247) of TC, which contains a putative antigenic site, was systemically deleted. Residues 1 to 240 was the minimum length of TC found to fold into an active conformation and reduced immunogenicity by 3-fold, but lead to a loss of RIP activity by about 10-fold [174]. Another Type I RIP, bouganin from the leaf of *Bougainvillea spectabilis* Willd. [175], was mutated to remove most T-cell epitopes, without affecting its biological activity, as compared to the wild-type molecule. This patented bouganin variant was genetically fused to an anti ephitelial cell adesion molecule (EpCAM) Fab fragment via a peptide linker containing a furin proteolytic site. resulting in the VB6-845 IT for treatment of solid tumors. VB6-845 IT was produced in *E. coli* by the Canadian company Viventia Biotechnologies Inc. and successfully tested on EpCAM-positive human tumor xenograft model in SCID mice [176]. A comprehensive series of pharmacology and toxicology studies have been conducted using VB6-845, including a phase I trial in patients bearing advanced squamous cell carcinomas of the head and neck. The results of this phase I trial showed that VB6-845 could reduce or stabilize tumors in 71% of patients with a maximum tolerated dose of 280 mg of IT, administered daily for five days. The IT was well tolerated with the only adverse effects being pain due to the intratumoral injections and reversibly elevated liver enzymes. These promising results warrant further clinical development of this plant RIP-based IT [177,178]. However, effects of the T-epitope removal on *in vivo* immune recognition are not yet being disclosed by the company.

A further complication observed in IT administration in the past was due to nonspecific binding of the toxin domain to vascular endothelial cells, leading to the so-called “vascular leak syndrome” (VLS) [179,180,181], which is characterized by interstitial edema, hypoalbuminemia, weight gain, and in most severe cases, pulmonary edema and hypotension. Although the mechanisms underlying this side effect are not completely understood, proteins such as RTA and some type I RIPs contain a consensus amino acid sequence “X-Asp-Y”, such as found in Interleukin-2, where X could be Leu, Ile, Gly or Val and Y could be Val, Leu or Ser, which seems to induce vascular damage to human endothelial cells *in vitro* by binding to integrin receptors [179,180,181] Indeed, in the case of RTA, molecular modeling suggested that these motifs were partially exposed on the surface of the molecule [179] and a similar motif is shared by viral disintegrins, which disrupt the function of integrin receptors [182]. In the perspective of eliminating VLS during therapeutical use of ricin-based ITs, Vitetta and coworkers produced a series of RTA mutants, and identified the Asn 97 to Ala mutation, in a region flanking the VLS-responsible motif in the three-dimensional structure, as displaying significant less VLS in mice [183]. Mutated RTA showing significantly less VLS in mice was also conjugated as an antiCD22 RFB4 IT and the latter could be injected in a five-fold higher concentration, as compared to the parental RFB4 IT [183]. Finally, connected to other unexpected toxicity issues, it is worth mentioning that a Phase I study with Combotox, a mixture of anti-CD19 and anti-CD22 RTA-based Its, led unfortunately to two drug-related deaths following treatment, due to problems with undetected protein aggregation [184]. This highlighted potential unforeseen serious problems with biological therapeutics such as IT preparations and indicates that every precaution must be taken to ensure the stability of these therapeutic molecules [109].

## 8. Complementary Approaches

### 8.1. Proof-of-Concept IT Potential Evaluation with Streptavidin-Saporin

Among potentially interesting complementary approaches, especially as a proof-of-concept before developing and producing a fully recombinant IT, is the use of Streptavidin-saporin to prepare conjugates with biotinylated targeted monoclonal antibodies, as proposed by Advance Targeting Systems, a company which also previously developed secondary antibodies conjugated to saporin as a means to identify the better internalizing target monoclonal antibodies [109]. This approach has been successfully used recently with a humanized monoclonal antibody to the prostate specific membrane antigen (PSMA), in order to evaluate IT antitumor activity against prostate carcinoma cell lines and *in vivo* as well. Cell surface binding was equivalent to the non-conjugated parental antibody and internalization took place specifically in PSMA positive cells but not in PC3 negative control cells. The IC_50_ varied from 0.1 nM to 100 nM depending on the cell line tested while the LNCap model cell line was the most sensitive with 60% of the treated cells undergoing apoptosis after 72 h. In a xenograft mouse model of LNCap cells, the IT showed potent *in vivo* anti-cancer activity [185]. Retuximab-saporin, an IT targeting human epidermal growth factor receptor (EGFR)-expressing cells, was obtained using the chimeric murine-human IgG1 monoclonal antibody Cetuximab (also termed C225 or “Erbitux”) bound via the biotin-streptavidin linkage to saporin. Photochemical internalization was also used in the latter case to enhance the cytotoxicity of the IT, by favoring its release from the endo-lysosomal compartments [186]. 

### 8.2. IT Potentiation Approaches

Saponins are low triterpenoid compounds mainly produced by plants, including *S. officinalis*. They affect plasma membrane permeability of living cells or artificial membranes by interacting with cholesterol and have been shown to greatly enhance saporin cytotoxicity towards several different cell lines [187,188,189,190]. It was demonstrated that cytotoxicity can be retained together with target specificity of a chimeric toxin, thus allowing the broadening of the therapeutic window with a simultaneous dose lowering. This saponin-mediated enhanced cytotoxicity was affected by drugs interfering with clathrin-mediated endocytosis, while inhibitors of caveolae-mediated endocytosis showed no effect and, furthermore drugs known to interfere with endo-lysosomal trafficking could also block saponin-mediated effects [33]. Indeed, recent data would suggest saponins treatment may be favoring endosomal escape of saporin [188]. A saporin fusion to epidermal growth factor via a cleavable adapter was constructed to evaluate its efficacy in inhibiting tumor growth and reduce side effects *in vivo*. In fact, a lethal dose for BALB/c mice was three-times less for the adapter-containing toxin (SA2E) than for the adapter-free construct (SE) [119]. Furthermore, SE only reduced the average weight of induced tumors by 33% whereas SA2E-treated mice exhibited a 71% reduction showing an almost complete suppression in 60% of the cases. Additionally, combined application of saponins increased cytotoxicity of SA2E on murine TSA tumor cells transfected with human EGFR by almost 20,000-fold using non permeabilizing concentrations of saponins [189]. Cervical cancers are the second most common cancers in women and since EGFR is found over-expressed in almost 90% of cervical tumors, targeting these receptors seems a promising approach: a combination of EGF-saporin SE fusion toxin with saponins resulted in a drastic enhancement of cytotoxicity ranging from 9,000-fold to 2,500,000-fold, again depending on the cell lines analyzed. The cytotoxicity of the fusion toxin was clearly target receptor specific because free EGF could effectively block this effect and, moreover, for all the cervical carcinoma cell lines tested, a clear correlation between EGFR expression and sensitivity to the SE fusion toxin could be observed [190]. Lipopolyamine and DMSO treatments have also been shown to significantly potentiate saporin and a urokinase-saporin conjugate [75]. Other such non toxic compounds/enhancers able to synergize IT administration would be welcome, such as the promising exponentially growing research into nano delivery particles whose size, shape, density and surface chemistry may dominate convective transport in the bloodstream, favor selective cellular uptake, as well as sub-cellular trafficking and localization, allowing greater efficacy and less side effects [191]. 

### 8.3. Non Viral Gene Delivery Approaches

Hoganson and coworkers [192] have used basic-FGF mediated targeting of FGF-2 receptor-bearing cells with either a DNA encoding cytosolic saporin or, for comparison, with one encoding the cytotoxic gene of herpes simplex virus thymidine kinase which is able to efficiently kill target cells by activating the ganciclovir prodrug and is so far the most widely used suicide gene in cancer gene therapy approaches. When the investigators compared FGF2-mediated delivery of a mammalian codon-optimized saporin gene to thymidine kinase DNA (followed by ganciclovir treatment) in tumor target cells, they resulted in a 60% and 75% decrease in cell numbers, respectively [192]. The driving idea is targeting tumor cells *in vivo* by means of synthetic carriers such as polycations in which selected binding peptides have been incorporated in order to trigger internalization of the DNA complexes within target tumor cells. This system has been patented [193] by Selective Genetics and covers the possibility to use several DNAs, including the Type I RIPs gelonin and saporin, and could be well included in the new frontiers of promising exponentially growing nanomedicine approaches [191,194,195,196]. *In vivo* delivery of genes encoding saporin or other Type I RIPs, might therefore also have great therapeutic potential for solid tumor treatments, as well. Indeed, we demonstrated that the intra-tumoral delivery of as low as 50 ng of a plasmid DNA encoding cytosolic saporin in an animal model, bearing an aggressive tumor such as B16 melanoma, could significantly retard tumor growth [53] and this was clearly due to the effects of a catalytically active toxin. A book dedicated to cancer gene therapy approaches includes also a chapter detailing preparation of cost-effective non-viral vectors loaded with the plant toxin gene for anticancer suicide gene therapy [196]. An intriguing observation we made was the presence of necrotic lesions at the injection site of B16 melanomas when using the native wild type saporin DNA but not a KQ inactive mutant [53], suggesting this might be due to secondary effects mediated by active saporin [53,196], which could partly be due to bystander effects by T-Killer cells. Interestingly, a similar observation was made when using a Plasminogen activator inhibitor-Saporin conjugate (which has been patented by Jankun and Hart as a method to deliver medicaments to cancer cells [197]). The conjugate, which was evaluated in a fibrosarcoma model in SCID mice and injected intratumorally, showing an effective antitumor response on mice with no systemic toxicity, revealed the presence of necrotic foci at the tumor site following administration. These intriguing observations deserve further investigations and these straightforward, easy and cost-effective cancer gene therapy approaches should be investigated for other Type I RIPs. These are potentially useful not only for accessible tumors but also after surgical removal of primary tumors for minimal residue killing of potentially harmful cells. 

## 9. Conclusions and Perspectives

Out of the 2485 entries retrieved in the Public NIH medical library database when entering “ribosome-inactivating protein” as a key word, half included ricin A chain or saporin, indicating the broad scientific interest these two plant RIPs have gained over the past years. While Ricin A chain has been explored more in targeted immunotherapy approaches for treating oncologic patients, highlighting some critical problems such as VLS that could be in part overcome [179,180,181,182,183], the worse problems faced were aggregation-prone preparations of RTA-based ITs [184]. In the case of saporin and other Type-I RIPs, less has been done and there are still several options to be pursued to optimize their therapeutic use (which could also be of interest to pharma companies). For instance, an Amgen patent covering single chain antibodies directed against deletion mutants of EGFR vIII and their therapeutic uses, also extended the patent to conjugation of the latter scFvs to several toxins selected from a group of different ones that also includes the plant RIP saporin [198]. 

Better design of fusion molecules, depletion of immunodominant epitopes, removal of sequences suspected of triggering VLS might give us the opportunity to fully exploit Type I plant toxins. Finally, the use of eukaryotic hosts for the large scale expression of the chimeric molecules will help to avoid misfolding and aggregation problems. Last but not least, the study of intracellular routes used by the parental toxins and the derived hybrid molecules would also help in broadening the use of agents specifically potentiating cytosolic delivery of these chimeric toxins. Amongst the most promising approaches are the local IT applications [113,145,166,177,178,197] (which may also help in avoiding undesirable side effects due to the systemic administration) in accessible tumors, such as head and neck, or for the minimal residue eradication of tumor cells, after surgical removal of the primary cancer. On the other hand, a careful selection of patients would be highly desirable for higher success rates to be achieved, to possibly avoid chemiotherapy regimens for those patients that could be predicted to resist these first choice treatments. A major challenge is still represented by the identification of early markers of the diseases, prognostic markers [136,137,138,139] for positive clinical responses to these therapies, and a particular focus in studying the mechanism(s) underlying different patients responses to immunotherapy in order to determine optimal selection criteria [99].
